# The fourth dimension of tool use: temporally enduring artefacts aid primates learning to use tools

**DOI:** 10.1098/rstb.2012.0410

**Published:** 2013-11-19

**Authors:** D. M. Fragaszy, D. Biro, Y. Eshchar, T. Humle, P. Izar, B. Resende, E. Visalberghi

**Affiliations:** 1 Department of Psychology, University of Georgia, Athens, GA 30602, USA; 2 Department of Zoology, University of Oxford, South Parks Road, Oxford OX1 3PS, UK; 3 DICE, School of Anthropology and Conservation, University of Kent, Marlowe Building, Canterbury CT2 7NR, UK; 4 Department of Experimental Psychology, University of São Paulo, Avenida Professor Mello Moraes, 1721, São Paulo, São Paulo 05508-030, Brazil; 5 Institute of Science and Technology of Cognition, National Research Council, Via Aldrovandi 16b, Rome 00197, Italy

**Keywords:** niche construction, artefact, tools, expertise, *Sapajus libidinosus*, *Pan troglodytes*

## Abstract

All investigated cases of habitual tool use in wild chimpanzees and capuchin monkeys include youngsters encountering durable artefacts, most often in a supportive social context. We propose that enduring artefacts associated with tool use, such as previously used tools, partly processed food items and residual material from previous activity, aid non-human primates to learn to use tools, and to develop expertise in their use, thus contributing to traditional technologies in non-humans. Therefore, social contributions to tool use can be considered as situated in the three dimensions of Euclidean space, and in the fourth dimension of time. This notion expands the contribution of social context to learning a skill beyond the immediate presence of a model nearby. We provide examples supporting this hypothesis from wild bearded capuchin monkeys and chimpanzees, and suggest avenues for future research.

## 1. Introduction

Tool use among wild animals holds interest for many scientists concerned with the origins and maintenance of skilled behaviours. In recent decades, attention has focused on the relationship between sociality and technical skills, as epitomized by the designation of various forms of tool use as traditions in social groups of non-human species, and even as definitional elements of culture in apes [1–4] and dolphins [5]. By definition, traditions require social support for their maintenance; new members of the group learn traditional skills in part through socially biased learning [6,7]. Socially tolerant, cohesive social systems are thought to be particularly favourable for socially biased learning [8–10]. Thus evolutionary explanations for the uneven appearance of technical traditions across species, time and place have focused on the evolution of social systems in which traditions are likely to develop, as well as on the evolution of cognitive skills relating to reasoning about physical processes, means–ends understanding and planning [11–14] and on the intersection of these skills and social systems [15–17].

We propose a complementary perspective here, framed in niche construction theory (NCT) [18,19], that highlights how residual artefacts of others' activity support learning technical skills. NCT posits that organisms, through their activities and choices, modify their own habitats and resources. Thus, individuals actively influence natural selection (on themselves and sometimes on individuals of neighbouring species) through behaviour, rather than passively being subject only to selection pressures arising in the environment [20]. NCT provides a basis to integrate the biological and social aspects of the behavioural sciences and is therefore generating a great deal of interest [21].

Particularly when individuals produce enduring changes in the environment, their activities result in ‘intergenerational persistence’ [20] of constructed niches. One manner in which this happens is through maintenance of a heritable ‘ontogenetic niche’ [22], a type of ‘ecological inheritance’ [15]. For many animals, the ontogenetic niche prominently features social partners, such as parents, siblings and group members; it is a constructed social niche, in the terminology of NCT. The constructed social niche underlies traditions, because the social setting in which young animals develop affects whether they will learn skills and habits characteristic of other members of their social group, and thus, the traditions they will acquire. In humans, this is recognized as the cultural niche ([20,23] see also [21,24]).

Altered physical features are also recognized in NCT as components of constructed niches. Indeed, the ‘poster child’ of NCT is the beaver, whose engineering works in dam building produce long-term consequences for itself and its immediate group, as well as for the local ecosystem. The physical dimension of the constructed niche is extremely rich in humans. Children grow up surrounded by clothing, furniture, shelters, musical instruments, and tools for food preparation, agriculture, hunting, personal hygiene, etc. They participate in the use of these artefacts as they become able to do so. The omnipresent constructed physical environment contributes to children learning technical skills, most powerfully in joint activity with other individuals [25]. Thus, the physical and social dimensions of the human-constructed ontogenetic niche complement one another. Human cultural evolution represents an extreme case of complementary physical and social niche construction [23,26,27].

With respect to non-human animals learning traditional skills, observation of another's action in real time is usually posited as the most powerful social influence, as exemplified by the phrase ‘demonstrator and observer’ commonly used in descriptions of social learning and designs of experiments on this topic [28] despite the fact that visual attention by the observer to the behaviours of the demonstrator is rarely quantified. The products of others' activity have also been posited to be a source of support for social learning of certain skills [29–31]. Experimental studies showed that encountering products, in the absence of others demonstrating the behaviour which produced the products, does support learning to access mechanically difficult foods (e.g. rats removing pine seeds from cones [32]; tits opening bottle caps from milk bottles [33]). Japanese macaques living in groups with stone-handling traditions (present in several areas of Japan [34]) preferentially handle stones that they encounter in piles, a by-product of others' prior stone-handling activity, over stones randomly scattered in the landscape [35]. Leca *et al*. [34,35] conclude that stone-handling is a ‘socially-induced behaviour’ where the artifacts of others' stone-handling activities play an important role in inducing the behaviour in others.

The role played by physical components (i.e. artefacts) in the maintenance of technical traditions, particularly tool use, in non-human animals has been overlooked, particularly for wild populations. To remedy this oversight, we propose that enduring artefacts associated with technical activities scaffold individuals' learning these skills in non-human species, and thus promote the maintenance of technical traditions, much as they do in humans (see also [19]). This constitutes an expansion of the traditional view of social learning in non-human animals that highlights immediate influences of social context derived from observation of action [36]. Here, we define ‘artefact’ as an object that is modified in some way by use, such as being placed in a specific location or position, or acquiring an odour, and ‘enduring’ as lasting long enough, while in an accessible location, for another individual to handle the artefact. This definition is looser than that used by archaeologists where artefact implies manufacture. In our view, enduring artefacts are of particular value in learning to use tools in feeding, the most common context in which tools are used by non-human primates. Artefacts support practice when others are not present at the site (thus reducing competition, which is a common feature in feeding contexts) and guide selection of materials and location by providing an example. Thus, they broaden temporally and spatially the opportunities for guided practice. For example, artefacts associated with tool use, such as previously used tools, partly processed foods items and residual material from previous activity may all serve this role.

Learning to use tools involves managing the multiple degrees of freedom involved in generating the correct forces, trajectories and orientations that the tool makes with objects and surfaces, and to do this skilfully takes considerable practice [37,38]. Simply observing another using a tool is not enough for even an adult novice to use a tool skilfully, and youngsters face much steeper challenges than adults in handling objects skilfully. Using most common hand tools takes humans years of practice to master (for example, scissors or cutlery). Managing multiple degrees of freedom inherent in using tools is more challenging for non-human primates than for humans [19,39]. Thus, we should expect that they need more practice than humans to master a similar problem. Accordingly, situational features that motivate individuals to handle the relevant materials in the right manner and in the right place may be particularly helpful for non-human primates to learn to use a tool skilfully.

In considering the importance of supporting practice, it is worth noting that persistent practice is regarded as essential to developing expertise in humans [40]. Helton [41] shows that developing expertise (defined as proficient performance according to pre-established criteria) takes about 10% of the lifespan across a wide range of species (for example, termite fishing by chimpanzees, *Pan troglodytes*, living in Gombe, Tanzania). Thus, technical skills such as tool use, which are at the boundaries of a species' behavioural capabilities, are expected to require a great deal of practice before the skill ‘pays off’ in delivery of benefits from performance.

In this report, we examine recent evidence in support of the hypothesis that enduring artefacts contribute to individuals' learning to use tools in non-human species by supporting persistent practice. Our data are drawn from two species of non-human primates that habitually use tools in natural settings: bearded capuchin monkeys (*Sapajus libidinosus*)^1^ and chimpanzees.

## 2. Artefacts and tool use in bearded capuchin monkeys

The EthoCebus research team studies how wild bearded capuchin monkeys living in the savannah-like Cerrado of Brazil (at Fazenda Boa Vista, hereafter FBV) crack tough palm nuts of several species (*Orbygnia* spp., *Attalea* spp. and others) using large stones as hammers and stone or log surfaces as anvils ([44,45]; http://www.EthoCebus.net). At FBV, most adults crack palm nuts using stone hammers routinely across the year [46]. Hammer stones weigh on average 1.1 kg [47]; adult monkeys weigh about 2 kg (females) or 3.7 kg (males) ([48]; D. Fragaszy, E. Visalberghi & P. Izar 2013, unpublished data). However, juveniles less than 5 years old rarely manage to crack a whole nut of the more resistant species (*Orbygnia* spp. and *Attalea* spp.). Aside from the physical challenge of lifting a heavy stone, nut-cracking presents daunting technical challenges such as placement of the nut in a stable position on the anvil surface, striking actions that hit the nut but do not displace it and are of sufficient force to crack the shell, controlling the stone throughout the striking cycle and catching the nut as it rolls following the strike so it does not fall off the anvil [48]. The monkeys use a bipedal stance during most of this activity, which is a challenging problem for dynamic balance [49]. Overall, it is not surprising that it takes young monkeys years to master nut-cracking. Recently, we have begun to study how they master this skill.

Young tufted capuchin monkeys (*Sapajus*) are generally free to approach and interact with all other group members without reprisal up to about 2 years of age, and even after that, tolerance among capuchin monkeys is marked [50,51]. However, adults face competition for access to anvil sites with hammer stones, and older juveniles are affected by this competition, receiving threats or being displaced from anvils by older individuals [52]. Thus, social dynamics are not uniformly supportive of youngsters accompanying adults at anvil sites. In any case, young capuchin monkeys watch their elders crack nuts with great interest, like the young capuchin monkeys in an urban park studied by Ottoni *et al*. [53]. Ramos da Silva [54] found that 25% of adults' cracking episodes at FBV were watched at close range by juveniles in the group, and in more than half of the watched episodes the juveniles scrounged bits of nuts or shells from the anvil. Nearly half of the time when juveniles scrounged, they did so while the adult was still present at the anvil (43% of scrounging events). This suggests that in the short-term, the infants' interest in adults' cracking activity is motivated by opportunities to find food. Nevertheless, we observed that youngsters devote considerable time to handling nuts, nutshells and stones, outside of opportunities to gain anything edible from these objects, when the group is in the vicinity of anvil sites. Youngsters also spend a good deal of time investigating artefacts: they ineffectively strike nuts and shells with other nuts and with stones, although usually with smaller stones than the adults use (because the latter are larger than the young monkeys can lift). These activities are not closely tied in time to feeding. Thus, interest in adults' ongoing activity, motivated by immediate interest in feeding, may be just one of several processes promoting young monkeys' investigation of cracking sites and their activity with artefacts related to cracking. It appears that artefacts are an integral part of the socially constructed niche in which young monkeys learn to crack nuts.

The development of nut-cracking has been studied in a population of tufted capuchin monkeys ranging freely in Tietê Park, a large urban park [55]. These monkeys crack the nut of *Syagrus* palms, which are much smaller and less resistant to cracking than the nuts cracked by the monkeys at FBV. Actions combining nuts and stones appeared after 1 year of age, in line with the timeline for appearance of vigorous actions combining objects in captive monkeys [56], but the monkeys did not crack nuts for several months after they began to strike objects placed on an anvil surface. Indeed, placing nuts on the anvil was the last component of nut-cracking to appear; simple percussion (of an object against a substrate) was the first. The late appearance of placing nuts suggests that releasing an object which capuchins valued was difficult to learn; their natural inclination is to keep a firm grasp on a potential food item. The two monkeys that succeeded at cracking nuts first did so at 19 and 26 months, although the elements of nut-cracking appeared in their repertoire many months earlier. The monkeys at FBV begin to crack broken pieces of nuts of resistant species of nut at about 2–3 years of age, roughly on the same timeline as the monkeys in Tietê Park, although in FBV, they cannot crack whole nuts for 1–2 more years, probably because these monkeys are cracking larger and more resistant nuts than the monkeys in Tietê Park.

As part of a study examining the role of artefacts and social context on capuchin monkeys' nut-cracking, the EthoCebus research team has collected continuous data on young monkeys' activity and location, and the concurrent activity, location and identity of neighbours, with particular attention to the materials and actions involved in cracking nuts. Here, we present findings from our first field season at FBV collecting what will become a longitudinal dataset on the form and timing of young bearded capuchin monkeys' activities related to nut-cracking. We observed 11 juveniles, aged 0.5–5 years, for 310 min each, on average (range 200–437 min), over an eight-week period in 2011. Methods are provided in the electronic supplementary materials, section 1.

**Figure 1.**
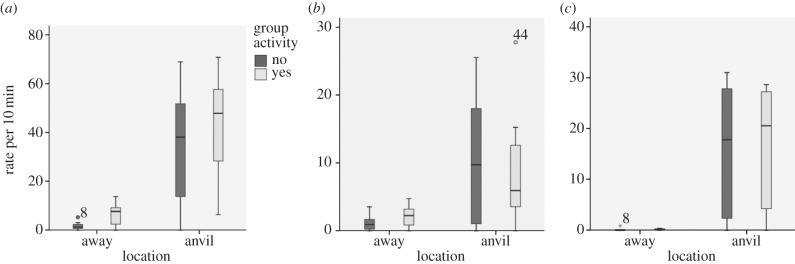
(*a*) Rate per 10 min for all actions with a nut, (*b*) all direct percussion of an object on a substrate and (*c*) all percussion of a nut with a stone by young capuchin monkeys while near anvil or away from an anvil, when no other group members were cracking or when one or more other group members were cracking. Panels (*a*,*b*) present data from 11 monkeys; (*c*) presents data from the eight older monkeys, as the youngest three monkeys never struck a nut with a stone. The median is represented by the dark bar, the interquartile range by the shaded box, and the maximum and minimum values by the whisker lines. Outliers are indicated by stars.

Young monkeys' attraction to artefacts associated with nut-cracking at FBV is patently obvious. For every variable we have examined, activities related to handling nuts and percussion occur at higher rates near an anvil than away from an anvil (figure 1). The effect of location is evident even when others are not currently cracking within the same minute. Linear regression analyses reveal a significant effect (*p* < 0.001) for location (near an anvil or away from an anvil) for all actions with a nut (**β** = 0.723), all direct percussion (**β** = 0.562) and striking a nut with a stone (**β** = 0.692). All three variables also showed a significant increase with age, with **β** ranging from 0.441 to 0.447 and all *p*-values < 0.001.The effect of concurrent activity by others, in contrast, was not significant for any of these variables (*p* = 0.098, all contact with a nut; 0.780, direct percussion and 0.884, striking a nut with a stone).

Given that nuts and nut shells are easily portable even for the youngest monkeys, that hard surfaces on which objects can be percussed are abundant throughout the monkeys' habitat [47,57], and that stones can be used to percuss objects other than nuts, the above results suggest that the sites and the artefacts present at these sites specifically promote activity relevant to cracking nuts. Thus, the activity of others cracking nuts and access to artefacts can synergistically promote strong and enduring interest in places and materials related to nut-cracking and support appropriate activities with these materials. It is difficult to specify exactly what youngsters learn about nut-cracking from persistent exploration and activity with artefacts, because it is years before they select nuts and stones and crack open nuts themselves. However, we expect that they practise motor skills associated only or primarily with nut-cracking (bipedal balance, placing nuts on the anvil and vigorous bimanual percussion) and learn through practice the affordances of the materials involved. For example, youngsters get extensive experience from direct manipulation with the resistant properties of nut shells long before they begin to crack them, and they practise placing nuts in pits on the anvils, which adults manage skilfully so that the more symmetrical side of the nut is placed facing the wall of the pit [58].

## 3. Artefacts and tool use in chimpanzees

Wild chimpanzees use tools in diverse ways across their geographical range [1,3]. We focus here on three forms of tool use seen routinely at Bossou, Guinea: cracking nuts with stones, collecting ants using a modified stick or stalk of vegetation (hereafter, ant dipping) and harvesting palm heart by pounding the centre of the oil-palm crown with a leaf frond (hereafter, pestle-pounding; for a recent comprehensive review of more than three decades of research at Bossou, see [59]). All three of these forms of tool use produce artefacts that can either be re-used by others shortly afterwards or in some cases, days or even months later depending on target resource availability and the durability of the material. Although most young chimpanzees will first start using tools to eat nuts, ants and palm heart during infancy (less than 4 years old), they require years of practice before achieving an adult level of proficiency [60,61]. The social contexts in which these foods are harvested differ, as we detail below, but in all three cases, we will argue, young are attracted to the artefacts produced by others, and their practice with these artefacts appears integral to their mastering the use of the relevant tool. The chimpanzees perform these tool-using activities regularly and in many areas of their home range.

### (a) Nut-cracking

The cracking of hard-shelled nuts by wild chimpanzees is restricted to West African populations [3], and variation among the communities studied has been reported in terms of the species of nut targeted, the objects chosen as tools and the precise techniques applied in hammering [62]. At Bossou, chimpanzees crack the oil-palm nut (*Elaeis guineensis*), the only naturally available nut species in the community's habitat. Chimpanzees typically nut-crack in the sitting position, placing nuts on the anvil one at a time and cracking them in a few strikes. Oil-palm nuts are locally abundant in the area near a fruiting tree, so many individuals can crack nuts concurrently if multiple hammers and anvils are present. Of note is the fact that Bossou is the only site at which chimpanzees use two separate, movable stones as hammer and anvil [63], rather than using anvils embedded in the ground as at all other sites studied (e.g. the Taï National Park; [64]). As a consequence, and unlike in the case of capuchin monkeys at FBV, both anvil and hammer stones can be re-used by another in another location or at the same location. Hammer stones at Bossou weigh on average 0.7 kg, and anvil stones 2.1 kg [65]. Even very young chimpanzees can pick up the hammer stones used by adults (although they cannot yet apply these with enough force to crack nuts)—in this respect, chimpanzees face a different challenge than do capuchins in learning how to crack nuts, because young capuchin monkeys cannot lift the stones used as hammers by adults. Long-term records indicate that while Bossou chimpanzees begin to manipulate objects involved in nut-cracking from an early age, no individual younger than 3.5 years has been seen to crack nuts successfully. In addition, no individual has been observed to learn to crack nuts after approximately 7 years of age, suggesting that there exists a sensitive period for learning this skill [62].

Young chimpanzees exhibit persistent interest in the nut-cracking activities of older group mates, and their attempts both to observe tool use from close range and to scrounge freshly cracked nuts are tolerated by related and unrelated adults [11]. Such tolerance wanes as the young mature: older juveniles are chased away increasingly frequently (which may in turn bring about the end of the sensitive period for learning this skill). Nonetheless, their ability to approach tools left behind by previous users is not restricted. Here, we compare, as a function of age, chimpanzees' choices of stones during nut-cracking sessions in terms of the objects' immediate previous history.

Data presented here were collected over a single field season (2002). At that time, seven of the nine adults (greater than 10 years), all four weaned young (5–10 years), and three of the five unweaned young (0–5 years) present were able to crack nuts. Observations were made at a natural clearing where experimenters provided ca 50 appropriately sized stones and several piles of oil-palm nuts [66]. All visits by chimpanzees to the experimental sites were video recorded, yielding 24.4 h of footage. The methods are further described in the electronic supplementary material, section 2a.

**Figure 2.**
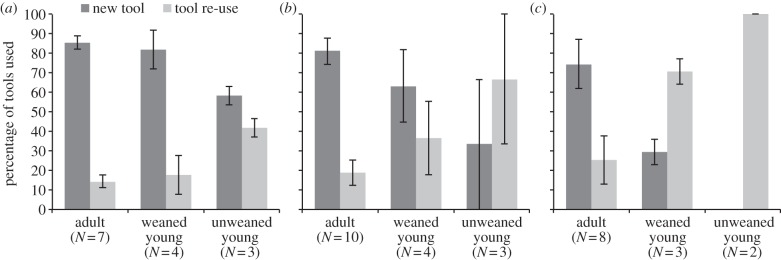
Sources of tools used by wild chimpanzees at Bossou, Guinea, as a function of the user's age (adult: over 10 years, weaned young: 5–10 years, unweaned young: 0–5 years) and the objects' previous history. ‘New tool’ refers to the use or manufacture of a tool not previously used by another individual; ‘tool re-use’ means that the individual is re-using an object previously used/made by another. The three panels correspond to three different forms of tool use: (*a*) Nut-cracking, (*b*) ant-dipping and (*c*) pestle-pounding. Only individuals that have been observed to perform the respective tool-use behaviours successfully are included in each dataset. Error bars are standard errors of the mean.

Each time a chimpanzee selected one or more stones for cracking, we recorded whether the same objects had previously been used during the same experimental session by other chimpanzees. Figure 2*a* shows that in unweaned young who had already learnt to crack nuts, just over 40% of stone selection involved re-use of another's recently used stones. Most frequently, these young re-used full hammer–anvil sets rather than just individual stones and used them at the vacated location, without moving the stones. In weaned young, on the other hand, the proportion of re-used tools dropped dramatically, to adult levels of less than 20% of stone selection episodes. During this period (years 5–10), the nut-cracking efficiency (measured as the average number of strikes needed to crack open a nut) of weaned young already begins to approach adult levels, although skills continue to be honed beyond the age of 10 [55].

The two youngest unweaned offspring are not included in figure 2*a*, as they had not yet learnt to crack nuts, but they did touch, roll and lift stones. Notably, despite the abundance of stones in the outdoor laboratory, these two infants only handled their mother's stones: tools that the mother had just stopped using, but had not yet moved away from. Both of these young were less than 1-year old, an age at which infants cling to and are carried by the mother at all times, and move only very short distances independently. During the earliest stages of learning, therefore, infants' access to stones is determined almost entirely by the mother's movements and tool activities.

### (b) Ant-dipping for army ants

Ant-dipping for army ants (*Dorylus* spp.) occurs in a very different physical and social context than nut-cracking [67,68]. Bossou chimpanzees target both army ants at the colony's nest (where the risk of being bitten is high) or while encountering opportunistically the ants travelling on the ground either hunting for prey or migrating to a new nesting site (where the risk of being bitten is low) [69]. Both woody or terrestrial herbaceous vegetation may serve in the manufacture of a suitable dipping tool [61]. When harvesting the biting ants at the nest, Bossou chimpanzees often damage the nest opening; the colony subsequently emigrates within 24 h and the nest site cannot then be re-used, unless the site is recolonized at a later date [70]. Ant-dipping site reutilization whether at nests or trails is extremely rare at Bossou, and therefore young chimpanzees infrequently have the opportunity to re-use tools left over by others between sessions, although discarded dipping tools can endure for days to months. Nevertheless, during the course of a session, individuals have the chance to re-use tools left behind by others. Weaned young are typically constrained competitively by the presence of adults but are highly motivated to practise ant-dipping. They practise ant-dipping significantly more often than unweaned young and tend to perform the behaviour generally for longer than unweaned young after adults have themselves ceased dipping [68]. Weaned young require several years of practice before acquiring an adult level of performance [50].

We report here data collected between June 2003 and March 2004 and July and September 2005 and 2006 at Bossou, Guinea from 17 chimpanzees ant-dipping across 40 sessions yielding 24 h of video recording. Methods are presented in the electronic supplemental material, section 2b.

Infants only engage in ant-dipping while their mothers are dipping [67]. Those mothers with dependent young (less than 5 years old) dip more often at trails than nests, which is less risky for the practising young. Youngsters of mothers who ant-dip frequently start observing and performing ant-dipping earlier than those whose mothers were classified as infrequent ant-dippers (age at onset of behaviour: 2.1 years versus 2.9 years, respectively) [67]. Young ones less than 5 years old use tools that were used previously by others two-thirds of the time. This proportion dropped to a third for weaned young (5–10 years old), while adults re-used another's tool during ant-dipping on average less than 20% of the time (figure 2*b*). In short, youngsters re-use adults' tools frequently during the lengthy period when they are honing their skills (5–10 years old).

### (c) Pestle-pounding

Pestle-pounding is unique to the Bossou community [71]. This behaviour, which requires bimanual coordination and forceful strikes, takes place at the top of the narrow crown of the oil palm. Therefore, in contrast to nut-cracking and ant-dipping, pestle-pounding provides limited opportunity for close observation by unskilled members of the group. This tool-use process requires several steps. First, the young fronds at the centre of the crown are removed and the petiole at the tip end of the frond is then consumed. Owing to the narrow space at the top of the crown, the other young fronds do not remain generally at the top but fall to the ground or are discarded once the petiole has been consumed. Ultimately, young frond removal provides access to the apical meristem but also to the raw material for a suitable pestle which is modified typically from one of the removed young fronds by shortening and/or removing the side leaflets. The pestle tool is then inserted into the access hole produced by the removal of the young fronds and the chimpanzee begins pounding at the palm heart. After each pounding action, the tool is laid aside and the chimpanzee inserts its arms into the hole to collect the mashed fibrous, juicy and sweet palm heart fibre.

We report here data collected between June 2003 and March 2004 and July and September 2005 and 2006 at Bossou, Guinea across 13 chimpanzees pestle-pounding on 32 separate occasions, yielding 24 h of video recordings. Methods are presented in the electronic supplementary material, section 2c.

Young chimpanzees can pull out young fronds on their own at a mean age of 6.9 ± 0.6 years (*N* = 3). Before then, youngsters depend on others to remove the young fronds and to provide access to the palm heart. Tool re-use is therefore necessary for youngsters who typically await the opportunity to take over a ‘free spot’ at the crown top of an oil palm. Chimpanzees less than 5 years old depend solely on re-used pestles typically previously used by their mother at the top of the crown and percentage re-use declines with age (figure 2*c*). Pestle and oil-palm re-use will generally yield a modest harvest, or even no harvest at all if the hole is already made too deep by previous user(s) for the fibrous material to be accessed. Nevertheless, young chimpanzees will still practise pestle-pounding with a tool left by another. After weaning it will take them years to perform the full behavioural sequence effectively on their own; until then, they have to depend solely on tool re-use to engage in pestle-pounding [50].

## 4. General findings

Across the studies with chimpanzees, we see two major parallels. First, youngest individuals re-use tools, and others' tolerance for young individuals allows them to be near others while they use tools. Infant chimpanzees are nearly universally tolerated and frequently obtain tools or manipulate materials relevant to tool-use activity while others are active at a tool-use site. Older juveniles typically have to wait for tools and tool-use sites to be abandoned before they can go there or retrieve the tools. Second, for tool-use behaviours that include a manufacturing phase (such as ant-dipping and pestle-pounding), younger individuals are less likely to manufacture a tool and more likely to use one previously used by another. With the proviso that capuchin monkeys transport their tools but do not manufacture them, a similar pattern appears with capuchin monkeys. Young monkeys are drawn to anvil sites, and while there, perform all the actions associated with nut-cracking. They perform these actions more often at these sites than elsewhere. Adults allow the youngest monkeys to do so freely; older juveniles must wait for their turn.

### (a) Contributions of artefacts to young individuals learning to use tools: are there general principles?

The social context in which youngsters explore the use of tools differs substantially between capuchin monkeys and chimpanzees, with broad influences by all members of the group in capuchin monkeys and with more nearly exclusive maternal or matrilineal influences in chimpanzees. Beyond the question of which individuals the juvenile may approach, however, both taxa share many features of an ontogenetic niche that we posit support learning to use tools through repeated practice. They both have lengthy juvenescence, are tolerated by adults, and are habitually co-present with adults at tool sites. As Helton [41] shows, acquiring expertise in a challenging task requires lengthy practice (he proposes 10% of the lifespan to acquire minimal expertise). His estimates match reasonably well with the timelines we have observed for the proficient mastery of various tool-using skills in capuchin monkeys and chimpanzees—up to 5 years in a lifetime of 30–50 years.

To our knowledge, habitual tool use in wild non-human primates always includes youngsters encountering durable artefacts, and most often encountering them in a supportive social context, that is while or soon after others have used the tools. All chimpanzee youngsters we have observed re-used stone tools, pestle tools, dipping sticks and leaf wads (used to drink water). All capuchin monkeys re-used anvils used by others, and as they became physically able to lift the stones, they re-used hammer stones left at the anvils. They often re-used pieces of nut as well, striking pieces of hard shell that may still contain edible material that can be loosened by percussive actions. Even though exploration and practice with artefacts by young individuals are inevitably dependent on task and circumstance, there are some general principles relevant to the importance of artefacts in learning to use tools. The same situation holds for another well-known tool-user in the animal kingdom, the New Caledonian crow [72]. Young crows follow their parents for several weeks after fledging, and scrounge food from them. They first exclusively use tools previously used by their parents, gradually manufacturing their own after many months. Thus, parents scaffold the young birds' learning to use tools. The parents' tool-use activity also provides an artefact in a way not present in non-human primates: the birds manufacture tools from the tough leaves of *Pandanus* trees by cutting and ripping segments from the leaves. Their actions leave an outline of the removed piece. These counterparts provide easier opportunities for young birds to rip a new segment than if they attempted to rip an intact leaf. This phenomenon suggests something to look for in other species: the possibility that manufacture of a tool creates an opportunity for another individual to manufacture another with less effort or with greater chances of success.

The data we have reviewed from non-human primates, bolstered with the example of New Caledonian crows, suggest the following as general principles of how artefacts can support young individuals learning to use tools:

#### (i) Adults’ tool-using behaviour scaffolds the physical circumstances in favour of youngsters handling appropriate objects in appropriate contexts through provision of durable artefacts

For example, in pestle-pounding, young chimpanzees simply do not have the strength to detach palm fronds. They rely 100% on used fronds left behind by adults. In nut-cracking, young chimpanzees and capuchin monkeys rely fully on the hammer stones transported by adults to anvil sites. Capuchin monkeys in particular cannot transport large stones, and these stones are rare in the landscape [57]. Although young capuchins at FBV practise percussion with a variety of materials that collect at anvil sites, appropriate and suitably large stones are present at the anvils for them to touch and smell and as soon as they are able to do so, they use them preferentially. Anvils contain pits, created from cracking nuts, where the adult monkeys preferentially place nuts to crack. The pits are also enduring artefacts that help youngsters master nut-cracking, because nuts placed in the pits are less likely to bounce off the anvil after being struck [48] and perhaps also for reasons concerning the direction of force on the shell of the nut when the stone strikes the nut.

Some tool-use tasks are particularly challenging for the young individual learning to use tools, and artefacts in these situations may be especially needed to provide support and guidance for practice. For example, tools used in ant-dipping and leaf-drinking are manufactured. Youngsters do not make their own tools for years, but in the interim, they re-use adults' tools. In these cases, artefacts are necessary for youngsters to practise at all.

#### (ii) Artefacts (when recognized by the learner as an artefact: as an item used by another) have positive affective value as well as physical affordances

The affective value of artefacts, derived from watching another using them, makes them powerfully attractive. For example, capuchin monkeys preferentially re-used the same pits as monkeys that cracked at an anvil before them [73]. Future studies should properly test this hypothesis by comparing the latency between seeing and using a tool by a young individual in two situations: when it sees a tool become available after another individual has used it versus when it sees a tool but sees no-one using it. Nut-cracking might provide an opportunity to do this, as the tools and the debris from their use are both long-enduring.

#### (iii) The objects’ physical affordances may be sufficient to promote appropriate actions, once the individual is motivated to handle them, in the absence of reinforcement

For example, sticks support probing, and stones support striking (and not vice-versa). Persistent practice of the appropriate action is prerequisite to developing expertise; powerful intrinsic motivation supports this practice. From this point of view, fidelity of copying the actions of another is not the basis for the persistence of technical traditions. Rather, features of the physical properties of the tools and of the tool activities that promote persistent practice in the absence of reinforcement hold the key to the development of technical traditions that require expertise. This argument differs from that of Matthews *et al*. [74] who showed that enhancement of interest followed by reinforcement was sufficient to induce durable behavioral variations in captive capuchin monkeys (see [75] for a similar example). What kinds of affordances lead to continued practice in the absence of reinforcement? Lockman [76] suggests that young individuals are intrinsically motivated to perform species-typical action routines (such as probing for chimpanzees and striking for humans and capuchins) and that exploratory performance of these routines supports the appearance of tool use in humans. We suggest the same is true for non-human species in which young individuals practise action routines that incorporate combining objects with other objects or surfaces, as is true for capuchins [56] and for chimpanzees [77].

#### (iv) Tool use is more likely to appear in situations that support the accumulation of artefacts and practice with artefacts than situations that do not

We predict that pestle-pounding is an uncommon form of tool use in chimpanzees, despite the abundance of appropriate palms, in part because the individual using a pestle tool often drops it to the ground after use, removing it from the site where others could practise with it. For dolphins in Shark's Bay, used sponges are swept away by the strong current running in the area where sponges are used to flush prey from the sandy sea floor, reducing their availability for re-use. Only a small portion of the population learns to use sponges in foraging [78]. Similarly, for capuchin monkeys, we predict that probing into arboreal insect nests is less common across populations than cracking nuts, because sticks used to probe would likely drop to the ground after use, whereas stones and nut debris typically remain near the anvil site when the tool-user leaves, and the typically embedded anvils remain in place.

In short, we propose that temporal durability of tools and debris (collectively, artefacts) from using tools and spatial durability of tool-use sites, coupled with socially mediated attraction to these artefacts and sites, and with the affordances of the artefacts for the performance of species-typical action routines, is particularly relevant for persistent practice (without successful performance) that underlies the development of technical expertise (*sensu* Helton [41]). These conditions are rare among non-human animals and consequently technological traditions requiring expertise are also rare [79,80] and particularly rare among aquatic species [81]. However, armed with this view, we can begin to search in a programmatic way for technological traditions involving tool use in new species and new situations. The key parameters concern social dynamics and physical setting of activity. For example, it will be interesting to see how artefacts are used by long-tailed macaques living on small islands off the coast of Thailand, studied by Gumert & Malaivijitnond [82]. These monkeys process a large variety of animal and plant targets using pounding tools, including scraping bivalves off rocks in the intertidal zone and cracking open loose molluscs on anvil sites. They are less tolerant to unrelated individuals, which may constrain youngsters' access to artefacts of others' cracking while the activity is occurring, compared to capuchins and chimpanzees. Nevertheless, we expect that young monkeys will make use of artefacts in some manner as they acquire tool-using skills. Ant-dipping also provides an opportunity to examine the consequences of artefacts on tool-use patterns. At Bossou, repeated ant-dipping at a nest site is rare, because once the driver ants have been severely disturbed they tend to move out. Where ant nest sites are re-used (e.g. Goualougo; [83]), we should find less variation in tool material selection, structure, length, etc., compared with Bossou, where ant-dipping tools more often are newly manufactured.

What is adaptive about this pattern of learning to use tools? We suggest, following the reasoning of integrative evolutionary scientists, that the learning process itself is an adaptation [23,84], rather than a particular skill. A learning process in which young individuals are persistently attracted to others' activity and the products of their activity, together with persistent motivation to engage in species-typical exploratory routines (*sensu* Lockman [76]) can support acquisition of diverse technical skills practised by others. Specific skills may have variable adaptive consequences within the lifetime of a particular individual, but across generations, the powerful and flexible learning process enabled by socially biased learning in physically constructed niches that encourage species-typical exploration will support the occurrence of locally appropriate skills. In this scenario, artefacts provide a spatio-temporal extension of social support for learning technical skills (the fourth dimension of our title).

## 5. Road map

- If artefacts play a role in learning to use tools, then we predict that tool use involving durable tools will be present in more populations than tool use involving ephemeral artefacts, because traditions of tool use producing more durable artefacts will be less susceptible to dying out over time. Surveys of published reports of tool use could test these proposals initially. New findings suggest that we will be able to study the temporal durability of stone tool-use traditions in some species of non-human primates from the study of their artefactual remains [85,86]. However, durable artefacts may be easier to find than ephemeral artefacts; we must diligently search for both kinds.
- It is important to determine whether or how artefacts (provided by others; i.e. social in origin) differ from other objects in what they afford for learning. These artefacts have been at the least *selected*, and possibly also *modified*/*manufactured* by others, and hence represent either a subset of available objects, or objects that are not even available in that form in the environment. In this case, artefacts do not merely afford opportunities for practice; they mould the practice.
- Artefacts may affect experts as well as learners. Re-use of hammers and some other kinds of tools is common in adult chimpanzees, for example, and thus can lead to homogeneity in tool use and selection. The role of artefacts as the foundation for homogeneity is relevant to paleoarcheologists considering the implications of tool styles as well as to behavioural scientists interested in the ontogenetic origins of individual and group characteristics. We urge researchers not to remove artefacts so as not to impact the learning opportunities of youngsters.
- Inheritance of artefacts as a component of the developmental niche may influence the likelihood and the pattern of cumulative culture in non-human animals, as it does in humans [23]. The social dynamics surrounding access to, and forms of activity with, artefacts, coupled with the pattern of cognitive development of the species in question, could either support or constrain the likelihood of young individuals modifying, and subsequently bequeathing to their peers and descendants, actions that produce artefacts. Canalization of activity through interaction with artefacts in the company of social partners may reduce variability of learned actions, as shown for example by Wood *et al*. [87] for young children.
- Developmental changes in the learning process deserve further detailed investigation. For example, does young monkeys' attraction to artefacts or to adults' activity change during development? On the adults' side, do adults modify their activity in accord with young individuals' interest in their activities in a way that may affect young individuals' practice?

These and many other questions about the role of artefacts in the acquisition of tool use and its maintenance as a traditional skill in a population are amenable to study in wild animals, as well as in captive settings. We hope to see further development and refinement of these ideas in the future.

## Endnote

1 Recent molecular analysis has revealed that capuchin monkeys, formerly identified as the single genus *Cebus*, are two genera, with the robust (tufted) forms now recognized as the genus *Sapajus* (including *libidinosus*, *apella*, *nigritus* and five other species), and the gracile (untufted) forms retained as the genus *Cebus* [42,43]. The nomenclature for *Sapajus* is registered with ZooBank (urn:lsid:zoobank.org:act:3AAFD645-6B09-4C88-B243-652316B55918). To date, tool use has been observed in some species of wild *Sapajus* but no species of wild *Cebus*.

## Data accessibility

Data have been deposited in the Dryad Repository: http://doi.org/10.5061/dryad.1m7sm.
